# Argonaute 2 Expression Correlates with a Luminal B Breast Cancer Subtype and Induces Estrogen Receptor Alpha Isoform Variation

**DOI:** 10.3390/ncrna2030008

**Published:** 2016-09-21

**Authors:** Adrienne K. Conger, Elizabeth C. Martin, Thomas J. Yan, Lyndsay V. Rhodes, Van T. Hoang, Jacqueline La, Muralidharan Anbalagan, Hope E. Burks, Brian G. Rowan, Kenneth P. Nephew, Bridgette M. Collins-Burow, Matthew E. Burow

**Affiliations:** 1 Vanderbilt University Medical Center, Department Medicine, Nashville, TN 37232, USA; adrienne.k.conger@vanderbilt.edu; 2 Department of Biological and Agricultural Engineering Louisiana State University Baton Rouge, LA 70803, USA; emart93@lsu.edu; 3 Department of Medicine-Section of Hematology and Medical Oncology, Tulane University, New Orleans, LA 70112, USA; tyan@tulane.edu (T.J.Y.); vhoang2@tulane.edu (V.T.H.); hburks@tulane.edu (H.E.B.); bcollin1@tulane.edu (B.M.C.-B.); 4 Department of Biological Sciences, Florida Gulf Coast University, Fort Myers, FL 33965, USA; lrhodes@fgcu.edu; 5 Department of Structural and Cellular Biology, Tulane University School of Medicine, New Orleans, LA 70112, USA; jla@tulane.edu (J.L.); manbalag@tulane.edu (M.A.); browan@tulane.edu (B.G.R.); 6 Medical Sciences and Department of Cellular and Integrative Physiology, Indiana University School of Medicine, Bloomington, IN 47405, USA; knephew@indiana.edu; 7 Department of Pharmacology, Tulane University, New Orleans, LA 70112, USA; 8 Tulane Cancer Center, Tulane University, New Orleans, LA 70112, USA

**Keywords:** AGO2, estrogen receptor, isoform, luminal B, miRNA

## Abstract

Estrogen receptor alpha (ERα) signaling pathways are frequently disrupted in breast cancer and contribute to disease progression. ERα signaling is multifaceted and many ERα regulators have been identified including transcription factors and growth factor pathways. More recently, microRNAs (miRNAs) are shown to deregulate ERα activity in breast carcinomas, with alterations in both ERα and miRNA expression correlating to cancer progression. In this study, we show that a high expression of Argonaute 2 (AGO2), a translation regulatory protein and mediator of miRNA function, correlates with the luminal B breast cancer subtype. We further demonstrate that a high expression of AGO2 in ERα+ tumors correlates with a poor clinical outcome. MCF-7 breast cancer cells overexpressing AGO2 (MCF7-AGO2) altered ERα downstream signaling and selective ERα variant expression. Enhanced ERα-36, a 36 kDa ERα isoform, protein and gene expression was observed in vitro. Through quantitative polymerase chain reaction (qPCR), we demonstrate decreased basal expression of the full-length ERα and progesterone receptor genes, in addition to loss of estrogen stimulated gene expression in vitro. Despite the loss, MCF-7-AGO2 cells demonstrated increased estrogen stimulated tumorigenesis in vivo. Together with our clinical findings on AGO2 expression and the luminal B subtype, we suggest that AGO2 is a regulator of altered ERα signaling in breast tumors.

## 1. Introduction

Breast cancer is one of the most common causes of death in U.S. women [1]. Subtypes of breast cancer, such as luminal A, luminal B, triple negative/basal-like, and HER2 type, are based on molecular characteristics including hormone receptor (estrogen receptor (ERα), progesterone receptor (PGR) and HER2/neu (V-Erb-B2 erythroblastic leukemia viral oncogene homolog 2)) status [2]. An estimated 70% of all newly diagnosed breast cancer patients are classified as ERα+ and hormone responsive, relying on estrogen stimulation to maintain tumorigenesis [3]. Alterations in estrogen signaling pathways in ERα+ breast tumors can arise from increased growth factor signaling, such as epidermal growth factor receptor (EGFR) and AKT/mammalian target of rapamycin (mTOR) pathways, and more recently to ERα isoform variation [2,4,5,6,7,8]. ERα-36 is a 36 kDa ERα isoform originating from within a conventional ERα transcript. Expression of ERα-36, which possesses DNA and ligand binding activity but lacks activation function-1 (AF-1) and -2 (AF-2) transactivation domains [4,8], has been observed in both ERα-positive and -negative breast tumors and cell lines [4,8,9]. ERα-36, which is localized to the plasma membrane and induces rapid estrogen signaling through mitogen activated protein kinase (MAPK) and phosphoinositide 3-kinase (PI3K)/AKT signaling pathways [8,10], is linked to tamoxifen resistance in breast cancers and is capable of eliciting agonistic tamoxifen induced signaling [8,9,10].

Recently, altered ERα signaling by microRNAs (miRNAs) has been observed, with some miRNAs directly repressing ERα and other miRNAs inducing resistance to endocrine therapies [11,12,13]. Deregulated miRNA processing and miRNA expression in breast tumor subtypes has also been reported [14,15,16,17,18]. In addition, the miRNA biogenesis associated proteins are associated with breast cancer progression. For example, Argonaute 2 (AGO2), a key component of the miRNA silencing complex and mRNA translational regulatory protein, is directly regulated by ERα signaling via the MAPK pathway [14]. Among breast tumor types, increased AGO2 expression in ERα− breast cancer cell lines and tumor samples has been observed [14]. A positive correlation between AGO2 expression levels and the ERα− phenotype in breast cancer cell lines and tumor samples has been previously reported with AGO2 expression being regulated by EGFR/MAPK signaling [14]. In this study we further evaluate the expression of AGO2 across breast cancer tumor samples with different degrees of receptor status (ERα, PGR, and HER2) and tumor molecular subtype. In addition, we show that a subset of ERα+ breast tumor samples, characterized as luminal B and ERα+/PGR−, demonstrated high AGO2 levels, similar to those observed in the ERα− tumor types. AGO2 expression correlates with a poor clinical outcome in ERα+ breast tumor samples.

## 2. Results

### 2.1. Enhanced Expression of AGO2 Is Associated with an ERα− and Luminal B Breast Cancer Phenotype

**Figure 1:**
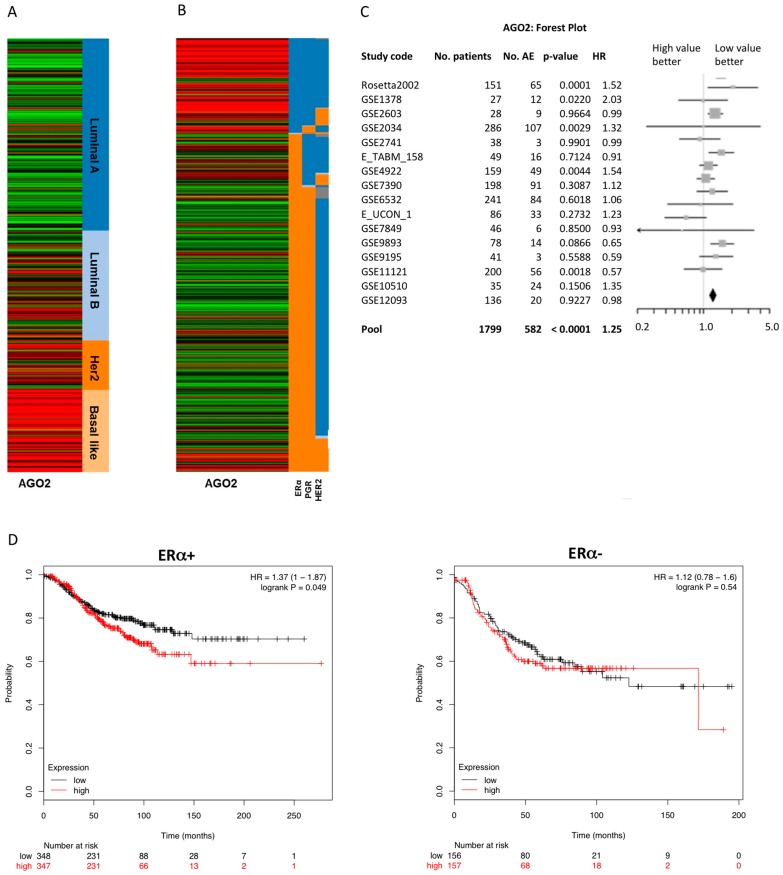
Argonaute 2 (AGO2) expression correlates with poor prognosis in estrogen receptor alpha positive (ERα+) breast cancer tumor samples. RNA deep sequencing of The Cancer Genome Atlas (TCGA) breast cancer samples were analyzed for AGO2 expression in correlation with (**A**) molecular subtype and (**B**) receptor status based on clinical scoring. Red indicates high gene expression levels and green indicates low gene expression. For receptors ERα, PGR, and HER2, orange shows positive and blue shows negative receptor expression; (**C**) Forest plot of univariate Cox analysis of AGO2 expression in breast tumor samples, correlation of AGO2 expression and clinical outcome; (**D**) Kaplan–Meier analysis of AGO2 expression in ERα+ and ERα− tumor samples with correlation with recurrence free survival for pooled breast cancer samples obtained from Breast Cancer Gene-Expression Miner v3.0. AE: number of events associated with survival, death or relapse; HR: hazard ratio.

Analysis of next generation deep sequencing of breast cancer invasive carcinoma gene expression was derived from The Cancer Genome Atlas (TCGA) data portal and viewed in the UCSC Cancer Genomics Browser [19,20,21,22]. AGO2 gene expression strongly correlated with the more aggressive and invasive basal-like breast cancer phenotypes and was inversely associated with ERα status (Figure 1A,B). Interestingly, tumor samples with a breast tumor type categorized as luminal B (Figure 1A) and an ERα+/PGR− receptor status (Figure 1B) demonstrated high AGO2 expression levels compared to samples with luminal A/ERα+/PGR+ receptor status. Clinically, luminal B tumor types can be categorized as having altered estrogen signaling pathways and an ERα+/PGR− phenotype [23]. To further determine the clinical significance of AGO2 expression in breast tumors, univariate Cox analysis was performed on breast tumor data obtained from the Breast Cancer Gene-Expression Miner V3.0 [24,25]. Increased AGO2 expression was associated with a negative clinical outcome for ERα+ but not for ERα− tumor types (Table 1). High levels of AGO2 significantly correlated with an increased hazard ratio and any event of relapse (AE) free survival (Figure 1C). Furthermore, Kaplan–Meier analysis of AGO2 expression correlated with a poor prognosis for recurrence free survival in ERα+ breast tumors but not in ERα− tumor samples (Figure 1D). These data illustrate an association between AGO2 expression in ERα+ tumors, and suggest AGO2 expression has a negative correlation with survival in ERα+ tumors.

### 2.2. Overexpression of AGO2 Represses Estrogen Signaling through Inhibition of ERα in Vitro but Does Not Diminish E2-Stimulated Tumorigenesis in Vivo

**Figure 2:**
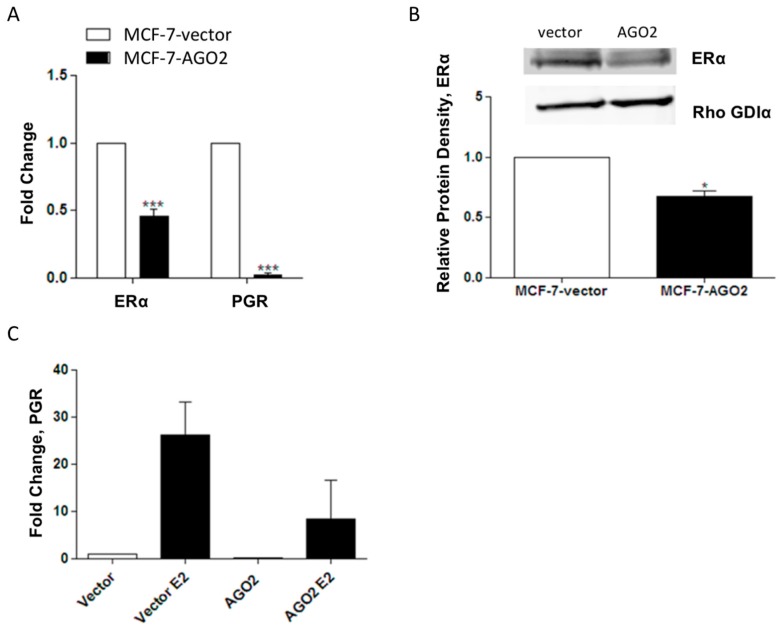
AGO2 expression suppresses ERα function in vitro. (**A**) MCF-7-AGO2 cell line was analyzed by quantitative PCR (qPCR) for ERα and progesterone receptor (PGR) expression levels vs. MCF-7-vector, *n* = 3 biological replicates; (**B**) Western blot for ERα protein levels in the MCF-7-AGO2 cell line, *n* = 3 biological replicates; (**C**) qPCR for PGR gene expression levels in MCF-7-AGO2 cell line vs. vector following 24 h of stimulation with 1 nM E2, *n* = 4 biological replicates. Significantly different * *p* < 0.05, *** *p* < 0.001.

To better understand the relationship between the AGO2 and ERα+ breast cancer phenotype, the ERα+ MCF-7 breast cancer cell line was stably transfected with a pIRES-AGO2 or pIRES-vector plasmid. MCF-7-AGO2 cells demonstrated a significant increase in AGO2 expression compared to MCF-7-vector cells both in mRNA and at the protein level (Figure S1A,B). To investigate the effects of AGO2 overexpression on estrogen signaling, we examined the expression of ERα and the ERα-regulated gene PGR in the MCF-7-vector and MCF-7-AGO2 cell lines. Basal ERα and PGR gene expression was decreased (Figure 2A) and ERα was suppressed at the protein level (Figure 2B) in the MCF-7-AGO2 cell line compared to MCF-7-vector. Repression of E2-induced stimulation of PGR was also observed (Figure 2C).

**Figure 3:**
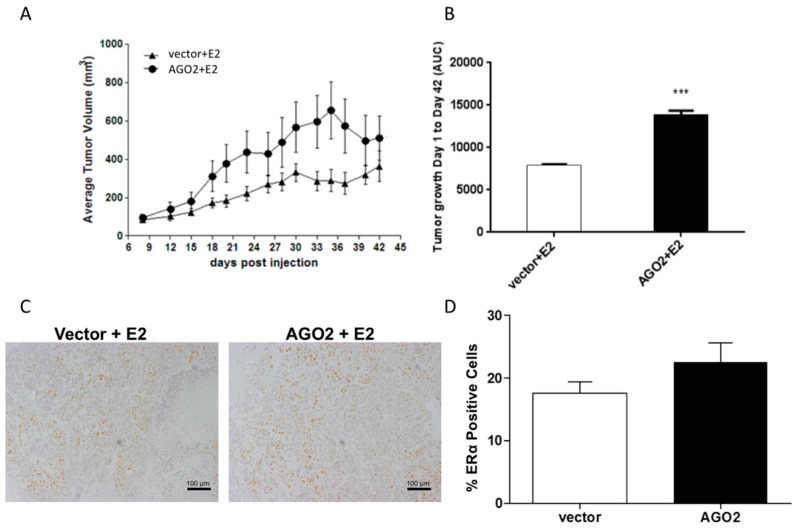
AGO2 enhances estrogen stimulated tumorigenesis in vivo. (**A**,**B**) Immunocompromised mice were inoculated with either 5 million MCF-7-vector or MCF-7-AGO2 cell lines in the presence of 60-day release estrogen pellet. (**A**) Results represent average tumor volume; (**B**) Results represent area under the curve (AUC). For all experiments error bars represent standard error of the mean (SEM), *n* = 10 for vector and *n* = 9 for AGO2 biological replicates. Significantly different *** *p* <0.001 (**C**) Tumors derived from MCF-7-AGO2 and MCF-7-vector inoculated mice were stained for ERα expression with immunohistochemistry, images obtained at 10× magnification. (**D**) Graphical representation of ERα staining from tumor samples, error bars represent SEM.

To determine if the changes observed in the MCF-7-AGO2 cell line in vitro translated to a similar response in vivo, ovariectomized SCID/Beige female mice were inoculated with either MCF-7-vector or MCF-7-AGO2 cells in the mammary fat pad in the presence of exogenous E2 (0.72 mg pellet, 60-day release, vs. placebo). E2 stimulated tumorigenesis was not inhibited by AGO2, and demonstrated significantly greater overall tumor growth kinetics as evaluated with area under the curve analysis (AUC) (Figure 3A,B) [26]. ERα protein expression was next evaluated in AGO2 tumors compared to vector. In our in vivo models there was no significant change in ERα protein expression as observed through immunohistochemistry staining (Figure 3C,D).

### 2.3. AGO2 Expression Selectively Increases ERα-36 Isoform Expression

**Figure 4:**
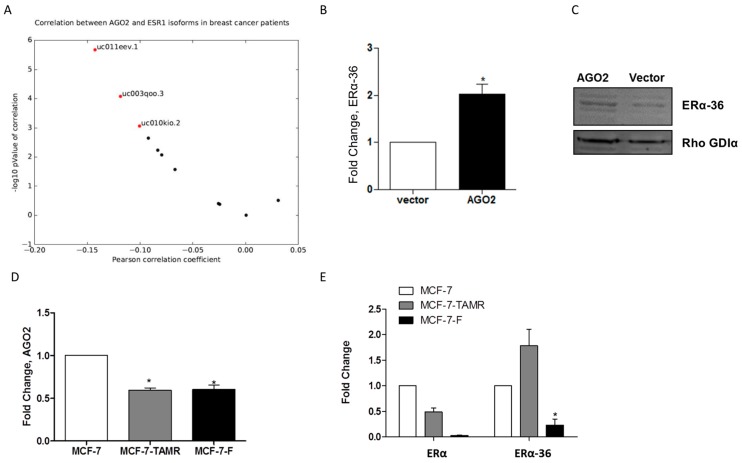
AGO2 expression selectively increases ERα-36 isoform expression. (**A**) qPCR for ERα-36 gene expression in the MCF-7-AGO2 cell line compared to vector and (**B**) Western blot for ERα-36 protein expression in the MCF-7-AGO2 cell line versus vector. Normalization was to Rho GDIα; (**C**) qPCR for AGO2 expression in MCF-7-parental, 4-OH tamoxifen resistant and ICI 182,780 resistant cell lines, MCF-7, MCF-7-TAMR and MCF-7-F, respectively (**D**) qPCR for ERα and ERα-36 gene expression in MCF-7-parental, 4-OH tamoxifen resistant and ICI 182,780 resistant cell lines, MCF-7, MCF-7-TAMR and MCF-7-F, respectively. Error bars represent SEM. Significantly different * *p* <0.05. All *n* = 3 biological replicates.

AGO2 increased E2-stimulated tumorigenesis in vivo and this may indicate that AGO2 mediates components of the estrogen signaling pathway. To identify possible mechanisms for the enhanced E2 response observed in vivo, we analyzed TCGA ERα positive breast cancer samples for a correlation of expression between AGO2 and ERα transcript gene expression. Of all evaluated transcripts, three ERα transcripts demonstrated a significant correlation with AGO2 expression. The most significantly correlated transcript with AGO2 gene expression was truncated three exon long ERα transcript (Figure 4A). Next, we examined expression of the truncated ERα splice variant ERα-36 in the MCF-7-AGO2 cell line. Quantitative polymerase chain reaction (qPCR) demonstrated that ERα-36 expression levels were significantly increased in the MCF-7-AGO2 cell line compared to vector control (Figure 4B). Western blot analysis for ERα-36 protein expression demonstrated increased ERα-36 expression in the MCF-7-AGO2 cell line versus vector (Figure 4C). ERα-36 is associated with tamoxifen resistance in breast cancer cells that exhibit resistance to endocrine therapies [10]. To further determine if a correlation exists between AGO2, ERα-36, and endocrine resistance, we next evaluated expression of AGO2 in the tamoxifen and ICI 182,780 resistant cell lines, MCF-7-TAMR and MCF-7-F respectively. Surprisingly, qPCR results demonstrated a loss of AGO2 gene expression in both the MCF-7-TAMR and MCF-7-F cell lines (Figure 4D). Evaluation of ERα and ERα-36 gene expression in these endocrine resistant cell lines demonstrated that ERα is repressed in both cell lines (in accordance with previously published data) and ERα-36 is deregulated in endocrine resistant cell lines [27]. ERα-36 demonstrated elevated expression in the MCF-7-TAMR cell line while it was repressed in the MCF-7-F cell line (Figure 4E). These data suggest there is not a universal correlation between AGO2 and endocrine resistance.

miRNAs are master regulators of many signaling pathways including those involved in ERα signaling. Since AGO2 is a known mediator of miRNA biogenesis, a miRNA gene array was performed to determine miRNA expression levels in the MCF7-AGO2 cell line compared to vector. As expected, several miRNAs were altered in the MCF-7-AGO2 cell line (Table S1). Interestingly, many of these miRNAs are known to either directly regulate ERα or enhance endocrine resistance [28]. These data suggest a possible mechanism for miRNA in the induction of an E2 response observed following overexpression of AGO2, however further investigations are warranted.

## 3. Discussion

Patients with luminal B and ER+/PGR− tumors have a poor response to endocrine therapies, including tamoxifen. The underlying mechanism appears to be deregulation in estrogen receptor signaling pathways [2,29], due to crosstalk of growth factor signaling pathways. PI3K/AKT/mTOR and the epidermal growth factor receptor (EGFR) crosstalk with ERα signaling to enhance pro-proliferative ERα regulated gene expression and suppress PGR gene expression [2]. AGO2, a key component of miRNA induced gene silencing, is regulated by the EGFR/MAPK signaling cascade and high AGO2 expression levels in ERα− breast cancers have been reported [14]. Here, we demonstrate for the first time, high AGO2 expression levels in a subset of ERα+ breast tumors (luminal B and ERα+/PGR−), similar to AGO2 expression observed in ERα− tumors. Furthermore, we show that high AGO2 gene expression levels in ERα+ tumors correlate with a poor prognosis, in contrast to a lack of correlation between AGO2 expression levels and clinical outcome for ERα− tumor types. Among ERα+ tumor types, loss of PGR expression correlates with aberrant estrogen signaling, and we show that AGO2 expression in an ERα+ breast cancer cell line can repress classical ERα signaling (loss of ERα expression and loss of E2 stimulation of PGR; Figure 2). Additionally, we show that despite the loss of classical estrogen signaling in vitro, estrogen-stimulated tumorigenesis is increased and there is no significant change. As a mechanism for altered ERα signaling, we next evaluated ERα-36 expression levels in the MCF-7 AGO2 cell line. AGO2 overexpression enhanced the expression of ERα-36 both at the gene and protein level. ERα-36 is involved in rapid estrogen signaling and its expression correlates with endocrine resistance. Surprisingly, MCF-7 generated with acquired endocrine resistance to tamoxifen (MCF-7-TAMR) and ICI (MCF-7-F) did not have enhanced ERα-36 gene expression. This study suggests that there may be a greater need in evaluating the alterations in miRNA biogenesis and associated genes in breast cancers demonstrating endocrine resistance.

## 4. Materials and Methods

### 4.1. Cells and Reagents

MCF-7 human breast cancer cell line was purchased from American Type Culture Collection (Manassas, VA, USA). MCF-7 and MCF-7-AGO2 validation of authenticity is provided as supplemental (Text S1 and S2 respectively). The MCF-7-TAMR and MCF-7-F cell lines were generated as previously described [27]. Cells lines were cultured as previously described [30]. Liquid nitrogen stocks were made upon receipt and maintained until the start of study. ERE-luciferase and/or qPCR for ERα and PGR were used to confirm MCF-7 sustained estrogen responsiveness. Morphology and doubling times were also recorded regularly to ensure maintenance of phenotype for all cell lines. Cells were used for no more than 6 months in culture. Cells were maintained in 10% fetal bovine serum (FBS) Dulbecco’s modified Eagle’s medium (DMEM) as previously described [31]. MCF-7 parental cells were thawed at passage 65 and were not used past passage 80. The MCF-7-AGO2 cell line was used at passage 7 to passage 25. 17β-Estradiol (E2) was purchased from Sigma-Aldrich (St. Louis, MO, USA).

### 4.2. Transfection of MCF-7 Cell Line

Parental MCF-7 cell line (passage 65) was stably transfected with pIRES-vector or pIRES-AGO2 plasmid (Addgene plasmid 10821 and 45567, Cambridge, MA, USA) with Lipofectamine 2000 per manufacturer’s protocol (Invitrogen, Grand Isles, NY, USA). Parental MCF-7 cells were grown in 100 mm dishes. The plasmid (5 μg) was added to 100 μL serum free opti-MEM followed by 15 μL Lipofectamine. After 30 min incubation, opti-MEM containing the plasmid was added. The following day, pIRES-transfected cells were treated with 200 ng/mL neomycin. Cells were grown in 10% DMEM and treated with 200 ng/mL neomycin every two days for 2 weeks. Colonies were pooled and verification of AGO2 overexpression was confirmed using qPCR. Stable pools of transfected cells were maintained in 10% DMEM as described above and were not used beyond passage 25.

### 4.3. RNA Extraction and Quantitative Real Time RT-PCR

MCF-7-pIRES-vector and MCF-7-AGO2 cells were harvested for total RNA extraction using Qiagen RNeasy ( Valencia, CA, USA). Quantity and quality of the RNA were determined by absorbance at 260 and 280 nm using the NanoDrop ND-1000. Total RNA (1 µg) was reverse-transcribed using the iScript kit (Bio-Rad Laboratories, Hercules, CA, USA) and qPCR was performed using SYBR-green and 300 ng cDNA (Bio-Rad Laboratories). β-Actin, PGR, ERα, and ERα-36 genes were amplified (*n* = 3) using the following primers: ERα forward 5′-GGCATGGTGGAGATCTTCGA-3′, ERα reverse 5′-CCTCTCCCTGCAGATTCATCA-3′, ERα-36 forward 5′-CAAGTGGTTTCCTCGTGTCTAAAGC-3′, ERα-36 reverse 5′-TGTTGAGTGTTGGTTCCAGG-3′, PGR forward 5′-TACCCGCCCTATCTCAACTACC-3′, PGR reverse 5′-TGCTTCATCCCCACAGATTAAACA-3′, β-actin forward 5′-TGAGCGCGGCTACAGCTT-3′, β-actin reverse 5′CCTTAATGTCACACACGATT3′. For E2 stimulation experiments, cells were grown in 5% DMEM for 48 h prior to 18 h of treatment with 1 nM E2. Data were analyzed by comparing relative target gene expression to β-actin. Relative gene expression was analyzed using 2^−ΔΔCt^ method. qPCR for miRNA was as follows: total RNA was extracted using the Qiagen miRNeasy kit as per the manufacturer’s protocol, small RNA fraction was not selected, 1.5 μg of total RNA was reverse transcribed using the Qiagen miscript II kit and qPCR was performed using miscript SYBR green and primers for U6 purchased from Qiagen. Normalization was to U6.

### 4.4. Western Blot

MCF-7-vector and MCF-7-AGO2 cells were grown in 10% FBS DMEM. Cells were washed with phosphate-buffered saline (PBS) and lysed with M-Per lysis buffer supplemented with 1% protease inhibitor and 1% phosphatase inhibitors (I/II) (Invitrogen). Supernatant containing protein extracts was obtained through centrifugation at 12,000 rpm (5415, Eppendorf, Westbury, NY, USA) for 10 min at 4 °C. Protein extracted per sample was determined by absorbance at 260 and 280 nm. Proteins were heat denatured and loaded on Bis-Tris-nuPAGE gel (Invitrogen). Protein transfer to nitrocellulose through iBlot and iBlot transfer stacks was per the manufacturer’s protocol (Invitrogen). Nonspecific binding was blocked by incubation in 3% milk (in 1% Tris buffered saline-Tween (TBS-T)) for 1 h. Overnight incubation of membrane with primary antibody for AGO2 diluted 1:1000 (Cell Signaling Technology, Beverly, MA, USA) and ERα (Santa Cruz Biotechnology, Santa Cruz, CA, USA) diluted 1:250, and ERα-36 diluted 1:500 at 4 °C followed by 3 × 15 min washes in 1% TBS-T. Membranes were incubated for 1 h in secondary antibody 1:10,000 dilution (LiCor Bioscience, Lincoln, NE, USA) followed by 3 × 10 min washes in 1% TBS-T. Band density was determined by LiCor gel imager. Normalization was to Rho GDI-α (Santa Cruz Biotechnology).

### 4.5. Animal Studies

Ovariectomized SCID/Beige female mice (4–6 weeks old, Charles River Laboratories; Wilmington, MA, USA) were allowed a 2-week period of adaptation in a sterile and pathogen-free environment with food and water ad libitum. Cells were harvested in the exponential growth phase using a PBS/ethylenediaminetetraacetic acid (EDTA) solution and washed. Viable cells (5 × 10^6^) in 50 µL of sterile PBS suspension were mixed with 100 µL Reduced Growth Factor Matrigel (BD Biosciences, Bedford, MA, USA). Injections were administered into the mammary fat pad using 27 ½ gauge sterile syringes. Animals were divided into treatment groups of five mice each: MCF-7 control vector, MCF-7 control vector plus E2, MCF-7 cells transduced to overexpress AGO2, MCF-7 cells transduced to overexpress AGO2 plus E2. Placebo or E2 pellets (0.72 mg of estradiol-17β, 60-day release; Innovative Research of America; Sarasota, FL, USA) were implanted subcutaneously in the lateral area of the neck using a precision trochar (10 gauge). All procedures in animals were carried out under anesthesia using a mix of isoflurane and oxygen. Tumor size was measured every 2–3 days using digital calipers. The volume of the tumor was calculated using the formula: 4/3π LS2 (L = larger radius; S = shorter radius). Animals were euthanized by cervical dislocation after exposure to CO_2_. Tumors were removed and frozen in liquid nitrogen or fixed in 10% formalin for further analysis. All procedures involving these animals were conducted in compliance with State and Federal laws, standards of the U.S. Department of Health and Human Services, and guidelines established by Tulane University Animal Care and Use Committee. The facilities and laboratory animals program of Tulane University are accredited by the Association for the Assessment and Accreditation of Laboratory Animal Care. (Protocol #: 4299R Approval Date: 3/8/2016)

### 4.6. Immunohistochemistry (IHC)

IHC was performed on 5 µm thickness sections made from paraffin-embedded tumor samples that were fixed with formalin 10% neutral buffered as described previously [32]. Briefly, slides with tumor sections were deparaffinized in xylene, dehydrated in ethyl alcohol, rinsed in water and antigen retrieval was done with Diva declocker for 30 min in a steamer and then incubated with 3% hydrogen peroxide for 5 min for quenching endogenous peroxides. The slides were rinsed with deionized water and PBS and then were blocked by incubation in 10% normal goat serum for 30 min. After blocking, the sections were incubated overnight with anti-ERα rabbit monoclonal primary antibody. The source of the primary antibody and the dilutions used for IHC are as follows: ERα (1:100; SP1 Thermo Scientific, Waltham, MA, USA). After overnight incubation with primary antibody, slides were washed with PBS followed by 30 min incubation with biotinylated secondary antibody (Vector Labs, Burlingame, CA, USA), rinsed in PBS and incubated with ABC reagent (Vector labs) for 30 min. Finally, 3,3-diaminobenzidine (DAB) was added to the sections and color was allowed to develop for 5 min and counterstained with hematoxylin for 30 s. Internal negative control samples incubated with either non-specific rabbit IgG, or 10% goat serum instead of the primary antibody showed no specific staining. Slides were dehydrated and mounted using two drops of Permount.

### 4.7. MicroRNA PCR Array

MCF-7 cells were plated at a density of 1 million cells in 25 cm^2^ flasks in normal culture media (10% DMEM) and allowed to adhere overnight at 37 °C. Cells were harvested in PBS, collected by centrifugation, and total RNA extracted using the miRNeasy kit (Qiagen) according to manufacturer’s protocol. Quantity and quality of RNA were determined by absorbance (260, 280 nm). SABiosciences Breast Cancer miRNA PCR array was used to detect changes in miRNA as per the manufacturer’s protocol and SABiosciences SYBR green (Qiagen).

### 4.8. Data Sources

TCGA research network breast cancer gene expression data (RNA-seq deep sequencing data) were viewed through the University of California, Santa Cruz (UCSC) Cancer Genomics Browser. The breast invasive carcinoma TCGA data set (total of *n* = 1032 tumor samples) was used and analyzed for gene expression aligned through the Illumina HiSeq system (Illumina, San Diego, CA, USA). Gene signatures were based on receptor status (ERα, PGR, and HER2) and molecular subtype (Luminal A, Luminal B, HER2-enriched, and basal-like). The linear relationships between AGO2 and ERα isoform RNA expression levels were measured in TCGA data sets for breast and invasive cancer. Pearson correlation coefficients and corresponding *p* values were calculated for each isoform with coefficients and −log10 transformed *p* values plotted on the X and Y axis, respectively.

Targeted analysis of prognostic gene expression for AGO2, amongst a cohort of breast tumor samples, was performed using the Breast Cancer Gene-Expression Miner v3.0. Table 1 designates ERα status, nodal status, and patient number. Kaplan–Meier analysis was performed from the “pool” of cohorts, meaning all data sets were merged from all studies and converted to a common scale with normalization. The prognostic impact of AGO2 was evaluated through the univariate Cox proportional hazard model obtained through pooled data [24,25].

## Figures and Tables

**Table 1:** AGO2 expression is inversely related to breast cancer clinical prognosis in ERα+ tumors but not ERα− tumors.

#	Population and Event Criteria ^1^			*p* Value	HR	95% CI	Good Prognosis RNA Level	No. Patients
1	N-	ERm	AE	<0.0001	1.25	1.15–1.36	Low	1799
2	Nm	ERm	MR	<0.0001	1.21	1.12–1.30	Low	2565
3	Nm	ERm	AE	<0.0001	1.16	1.10–1.24	Low	3300
4	N−	ER+	AE	<0.0001	1.30	1.17–1.45	Low	1388
5	Nm	ER+	MR	<0.0001	1.26	1.14–1.39	Low	1929
6	N−	ER+	MR	<0.0001	1.33	1.17–1.52	Low	1146
7	N−	ERm	MR	<0.0001	1.24	1.12–1.37	Low	1492
8	Nm	ER+	AE	<0.0001	1.18	1.09–1.28	Low	2483
9	N+	ERm	MR	0.0549	1.13	1.00–1.29	Low	842
10	N+	ER+	MR	0.1156	1.14	0.97–1.34	--	652
11	N−	ER	AE	0.1564	1.13	0.95–1.33	--	390
12	Nm	ER	AE	0.4186	1.05	0.94–1.17	--	774
13	N+	ER	AE	0.4618	0.93	0.77–1.13	--	226
14	N+	ER	MR	0.5316	0.92	0.72–1.19	--	173
15	Nm	ER	MR	0.6938	1.03	0.89–1.18	--	601
16	N+	ER+	AE	0.7713	0.98	0.86–1.11	--	815
17	N−	ER	MR	0.8048	1.02	0.84–1.24	--	330
18	N+	ERm	AE	0.8467	1.01	0.91–1.12	--	1058
11	N−	ER	AE	0.1564	1.13	0.95–1.33	--	390
12	Nm	ER	AE	0.4186	1.05	0.94–1.17	--	774
13	N+	ER	AE	0.4618	0.93	0.77–1.13	--	226
14	N+	ER	MR	0.5316	0.92	0.72–1.19	--	173
15	Nm	ER	MR	0.6938	1.03	0.89–1.18	--	601
16	N+	ER+	AE	0.7713	0.98	0.86–1.11	--	815
17	N−	ER	MR	0.8048	1.02	0.84–1.24	--	330
18	N+	ERm	AE	0.8467	1.01	0.91–1.12	--	1058

^1^ ER = estrogen receptor status, N = nodal status, and event (AE = any event, MR = metastatic relapse). HR: hazard ratio; CI: confidence interval.

## Supplementary Files

Supplementary File 1

## Abbreviations

The following abbreviations are used in this manuscript:

AF: activation function
AGO2: argonaute 2
EGFR: epidermal growth factor receptor
ERα: estrogen receptor
HER2/neu: V-Erb-B2 Erythroblastic Leukemia Viral Oncogene Homolog 2
MAPK: mitogen activated protein kinase
mTOR: mammalian target of rapamycin
miRNAs: microRNAs
PGR: progesterone receptor
