# Supplementary material for: Argonaute 2 Expression Correlates with a Luminal B Breast Cancer Subtype and Induces Estrogen Receptor Alpha Isoform Variation
Source: Noncoding RNA. 2016 Sep 21;2(3):8. doi: 10.3390/ncrna2030008 (PMC5831908; doi:10.3390/ncrna2030008)
Supplement: Supplementary File 1 [file ncrna-02-00008-s001.pdf]

Non-Coding RNA **2016**, *2*, 8; doi: 10.3390/ncrna2030008

Argonaute 2 Expression Correlates with a Luminal B Breast Cancer Subtype and Induces Estrogen Receptor Alpha Isoform Variation

Adrienne K. Conger, Elizabeth C. Martin, Thomas J. Yan, Lyndsay V. Rhodes, Van T. Hoang, Jacqueline La, Muralidharan Anbalagan, Hope E. Burks, Brian G. Rowan, Kenneth P. Nephew, Bridgette M. Collins-Burow and Matthew E. Burow

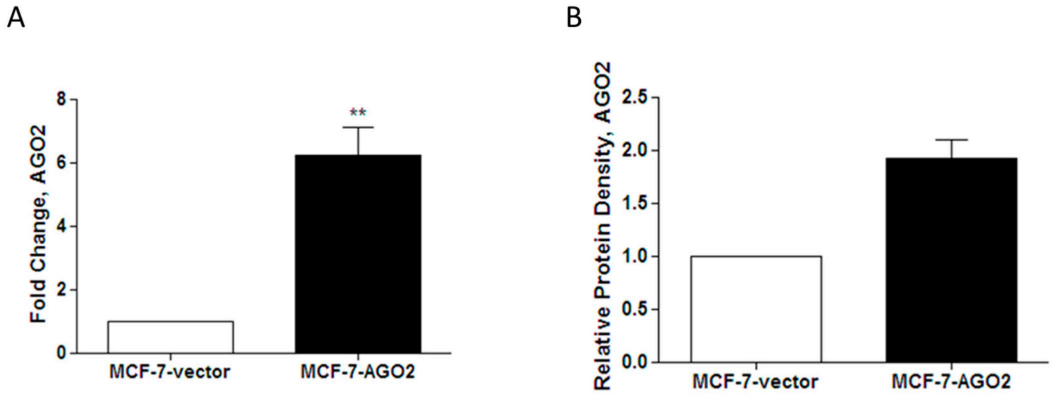

Figure S1. Validation for the overexpression of AGO2 in the MCF-7-AGO2 cell line. (**A**) qPCR and (**B**) Western blot for AGO2 gene and protein levels respectively in the MCF-7-AGO2 cell line vs. –vector. ** $p < 0.01$. $n = 3$ biological repeats.

Table S1. Overexpression of AGO2 alters the miRNA expression profile in the MCF-7 breast cancer cell line.

Expression Changes	miRNAs	Role	Target Genes	Refs.
Upregulated (>1.5 Fold Change)	hsa-miR-196a	Oncogenic	*ANXA1*	[1]
	hsa-miR-193b	Tumor Suppressor	*KRAS, MAX*	[2]
	hsa-miR-20b	Oncogenic	*HIF1A, MDR1, HIPK2, PTEN*	[3,4]
	hsa-miR-21	Oncogenic	*TGFB, TRIM2, SKI, RAS, RAB6A, RAB6C, PDCD4, GRHL3, PTEN*	[1,5-9]
	hsa-miR-335	Tumor Suppressor	*ROCK1*	[10]
	hsa-miR-132	Oncogenic	*RB1*	[11]
	hsa-miR-125a-5p	Tumor Suppressor	*BCL2, BCL2L12, MCL-1*	[1,12]
	hsa-miR-124	Tumor Suppressor	*ERK2*	[13]
	hsa-miR-212	Oncogenic	*RB1*	[11]
	hsa-let-7i	Tumor Suppressor	*RAS*	[14]
	hsa-miR-134	Oncogenic	*WWOX*	[15]
	hsa-miR-200c	Tumor Suppressor	*ZEB1, ZEB2*	[16]
	hsa-miR-18b	Tumor Suppressor	*Erα, MDM2-p53*	[17]
	hsa-let-7e	Tumor Suppressor	*RAS*	[14]
	hsa-miR-205	Oncogenic	*SHIP2*	[17]
	hsa-miR-215	Oncogenic	*RB1*	[18]
	hsa-let-7f	Tumor Suppressor	*MYH9*	[19]
	hsa-miR-184	Oncogenic	*SOX7*	[20]
Downregulated (<−1.5 Fold Change)	hsa-miR-210	Oncogenic	*ISCU*	[1,21]
	hsa-miR-96	Tumor Suppressor	*KRAS*	[22]
	hsa-miR-181d	Tumor Suppressor	*KRAS, BCL2*	[23]
	hsa-miR-100	Oncogenic and Tumor Suppressor	*RBSP3*	[1,24]
	hsa-let-7b	Tumor Suppressor	*RAS*	[1,14]
	hsa-miR-148a	Tumor Suppressor	*ROCK1*	[25]
	hsa-miR-29b	Tumor Suppressor	*TIAM1*	[26]
	hsa-miR-143	Tumor Suppressor	*COX2*	[1,27,28]
	hsa-miR-363	Tumor Suppressor	*PDPN*	[29]
	hsa-miR-29a	Oncogenic and Tumor Suppressor	*TCL1*	[1,30]
	hsa-let-7d	Tumor Suppressor	*RAS*	[14]
	hsa-miR-125b	Tumor Suppressor	*MMP13*	[1,31]
	hsa-miR-92a	Oncogenic	*CDH1*	[32]

Table S1. *Cont.*

Expression Changes	miRNAs	Role	Target Genes	Refs.
Downregulated (<−1.5 Fold Change)	hsa-let-7g	Tumor Suppressor	*RAS*	[14]
	hsa-miR-34a	Tumor Suppressor	*SIRT6*	[33]
	hsa-miR-25	Oncogenic	*BIM*	[34]
	hsa-miR-20a	Oncogenic	*TIMP2, ATG7*	[35]
	hsa-miR-135b	Oncogenic	*LATS2, β-TrCP, MDR2, LZTS1*	[1,36]
	hsa-miR-10b	Tumor Suppressor	*TIAM1*	[1,37]
	hsa-miR-140-5p	Tumor Suppressor	*VEGFA*	[1,38]
	hsa-miR-34c-5p	Tumor Suppressor	*BMF*	[39]
	hsa-miR-27a	Oncogenic	*HIF1A, MDR1, HIPK2*	[3]
	hsa-miR-218	Tumor Suppressor	*CDK6, RICTOR, CTSB*	[40]
	hsa-miR-98	Tumor Suppressor	*IL6*	[41]
	hsa-miR-181a	Oncogenic and Tumor Suppressor	*RaIA, ATM*	[42,43]
	hsa-miR-181c	Oncogenic	*WIF-1*	[44,45]
	hsa-miR-130a	Oncogenic	*PTEN, ZBTB4, TGFBR2, SMAD4*	[1]
	hsa-miR-191	Oncogenic	*BDNF, CDK6, SATB1*	[46]
	hsa-miR-32	Tumor Suppressor	*SOX9*	[47]
	hsa-miR-18a	Tumor Suppressor	*KRAS, CDC42*	[48,49]
	hsa-miR-15b	Tumor Suppressor	*BCL2*	[50]
	hsa-miR-149	Tumor Suppressor	*RAP1a, RAP1b*	[51]
	hsa-miR-301a	Oncogenic	*SMAD4*	[1,52]
	hsa-miR-17	Oncogenic	*PTEN, ZBTB4, TGFBR2, SMAD4*	[1]
	hsa-miR-7	Tumor Suppressor	*PIK3CD*	[53]
	hsa-miR-193a-5p	Tumor Suppressor	*PIK3R3, mTOR*	[54]
	hsa-miR-128	Tumor Suppressor	*BMI-1, NANOG, TGFBR1*	[55]
	hsa-miR-23b	Tumor Suppressor	*ZEB1*	[56]
	hsa-miR-9	Oncogenic	*LASS2*	[57]
	hsa-miR-19a	Oncogenic	*PTEN, ZBTB4, TGFBR2, SMAD4*	[1]
	hsa-miR-203	Tumor Suppressor	*c-JUN*	[58]

Supplementary References

1. Hamilton, M.P.; Rajapakshe, K.; Hartig, S.M.; Reva, B.; McLellan, M.D.; Kandoth, C.; Ding, L.; Zack, T.I.; Gunaratne, P.H.; Wheeler, D.A., *et al.* Identification of a pan-cancer oncogenic microrna superfamily anchored by a central core seed motif. *Nat Commun* **2013**, *4*, 2730.
2. Gastaldi, C.; Bertero, T.; Xu, N.; Bourget-Ponzio, I.; Lebrigand, K.; Fourre, S.; Popa, A.; Cardot-Leccia, N.; Meneguzzi, G.; Sonkoly, E., *et al.* Mir-193b/365a cluster controls progression of epidermal squamous cell carcinoma. *Carcinogenesis* **2014**, *35*, 1110-1120.
3. Danza, K.; Silvestris, N.; Simone, G.; Signorile, M.; Saragoni, L.; Brunetti, O.; Monti, M.; Mazzotta, A.; De Summa, S.; Mangia, A., *et al.* Role of mir-27a, mir-181a and mir-20b in gastric cancer hypoxia-induced chemoresistance. *Cancer Biol Ther* **2016**, 0.
4. Zhou, W.; Shi, G.; Zhang, Q.; Wu, Q.; Li, B.; Zhang, Z. Microrna-20b promotes cell growth of breast cancer cells partly via targeting phosphatase and tensin homologue (pten). *Cell Biosci* **2014**, *4*, 62.
5. Chan, J.A.; Krichevsky, A.M.; Kosik, K.S. Microrna-21 is an antiapoptotic factor in human glioblastoma cells. *Cancer Res* **2005**, *65*, 6029-6033.
6. Darido, C.; Georgy, S.R.; Wilanowski, T.; Dworkin, S.; Auden, A.; Zhao, Q.; Rank, G.; Srivastava, S.; Finlay, M.J.; Papenfuss, A.T., *et al.* Targeting of the tumor suppressor grhl3 by a mir-21-dependent proto-oncogenic network results in pten loss and tumorigenesis. *Cancer Cell* **2011**, *20*, 635-648.
7. Dziunycz, P.; Iotzova-Weiss, G.; Eloranta, J.J.; Lauchli, S.; Hafner, J.; French, L.E.; Hofbauer, G.F. Squamous cell carcinoma of the skin shows a distinct microrna profile modulated by uv radiation. *J Invest Dermatol* **2010**, *130*, 2686-2689.
8. Iorio, M.V.; Ferracin, M.; Liu, C.G.; Veronese, A.; Spizzo, R.; Sabbioni, S.; Magri, E.; Pedriali, M.; Fabbri, M.; Campiglio, M., *et al.* Microrna gene expression deregulation in human breast cancer. *Cancer Res* **2005**, *65*, 7065-7070.
9. Li, X.; Huang, K.; Yu, J. Inhibition of microrna-21 upregulates the expression of programmed cell death 4 and phosphatase tensin homologue in the a431 squamous cell carcinoma cell line. *Oncol Lett* **2014**, *8*, 203-207.
10. Liu, H.; Li, W.; Chen, C.; Pei, Y.; Long, X. Mir-335 acts as a potential tumor suppressor mirna via downregulating rock1 expression in hepatocellular carcinoma. *Tumour Biol* **2015**, *36*, 6313-6319.
11. Park, J.K.; Henry, J.C.; Jiang, J.; Esau, C.; Gusev, Y.; Lerner, M.R.; Postier, R.G.; Brackett, D.J.; Schmittgen, T.D. Mir-132 and mir-212 are increased in pancreatic cancer and target the retinoblastoma tumor suppressor. *Biochem Biophys Res Commun* **2011**, *406*, 518-523.
12. Tong, Z.; Liu, N.; Lin, L.; Guo, X.; Yang, D.; Zhang, Q. Mir-125a-5p inhibits cell proliferation and induces apoptosis in colon cancer via targeting bcl2, bcl2l12 and mcl1. *Biomed Pharmacother* **2015**, *75*, 129-136.
13. Yamane, K.; Jinnin, M.; Etoh, T.; Kobayashi, Y.; Shimozono, N.; Fukushima, S.; Masuguchi, S.; Maruo, K.; Inoue, Y.; Ishihara, T., *et al.* Down-regulation of mir-124/-214 in cutaneous squamous cell carcinoma mediates abnormal cell proliferation via the induction of erk. *J Mol Med (Berl)* **2013**, *91*, 69-81.
14. Johnson, S.M.; Grosshans, H.; Shingara, J.; Byrom, M.; Jarvis, R.; Cheng, A.; Labourier, E.; Reinert, K.L.; Brown, D.; Slack, F.J. Ras is regulated by the let-7 microrna family. *Cell* **2005**, *120*, 635-647.
15. Liu, C.J.; Shen, W.G.; Peng, S.Y.; Cheng, H.W.; Kao, S.Y.; Lin, S.C.; Chang, K.W. Mir-134 induces oncogenicity and metastasis in head and neck carcinoma through targeting wwox gene. *Int J Cancer* **2014**, *134*, 811-821.
16. Park, S.M.; Gaur, A.B.; Lengyel, E.; Peter, M.E. The mir-200 family determines the epithelial phenotype of cancer cells by targeting the e-cadherin repressors zeb1 and zeb2. *Genes Dev* **2008**, *22*, 894-907.

17. Dar, A.A.; Majid, S.; Rittsteuer, C.; de Semir, D.; Bezrookove, V.; Tong, S.; Nosrati, M.; Sagebiel, R.; Miller, J.R., 3rd; Kashani-Sabet, M. The role of mir-18b in mdm2-p53 pathway signaling and melanoma progression. *J Natl Cancer Inst* **2013**, *105*, 433-442.
18. Deng, Y.; Huang, Z.; Xu, Y.; Jin, J.; Zhuo, W.; Zhang, C.; Zhang, X.; Shen, M.; Yan, X.; Wang, L., *et al.* Mir-215 modulates gastric cancer cell proliferation by targeting rb1. *Cancer Lett* **2014**, *342*, 27-35.
19. Liang, S.; He, L.; Zhao, X.; Miao, Y.; Gu, Y.; Guo, C.; Xue, Z.; Dou, W.; Hu, F.; Wu, K., *et al.* Microrna let-7f inhibits tumor invasion and metastasis by targeting myh9 in human gastric cancer. *PLoS One* **2011**, *6*, e18409.
20. Wu, G.G.; Li, W.H.; He, W.G.; Jiang, N.; Zhang, G.X.; Chen, W.; Yang, H.F.; Liu, Q.L.; Huang, Y.N.; Zhang, L., *et al.* Mir-184 post-transcriptionally regulates sox7 expression and promotes cell proliferation in human hepatocellular carcinoma. *PLoS One* **2014**, *9*, e88796.
21. McCormick, R.I.; Blick, C.; Ragoussis, J.; Schoedel, J.; Mole, D.R.; Young, A.C.; Selby, P.J.; Banks, R.E.; Harris, A.L. Mir-210 is a target of hypoxia-inducible factors 1 and 2 in renal cancer, regulates iscu and correlates with good prognosis. *Br J Cancer* **2013**, *108*, 1133-1142.
22. Yu, S.; Lu, Z.; Liu, C.; Meng, Y.; Ma, Y.; Zhao, W.; Liu, J.; Yu, J.; Chen, J. Mirna-96 suppresses kras and functions as a tumor suppressor gene in pancreatic cancer. *Cancer Res* **2010**, *70*, 6015-6025.
23. Wang, X.F.; Shi, Z.M.; Wang, X.R.; Cao, L.; Wang, Y.Y.; Zhang, J.X.; Yin, Y.; Luo, H.; Kang, C.S.; Liu, N., *et al.* Mir-181d acts as a tumor suppressor in glioma by targeting k-ras and bcl-2. *J Cancer Res Clin Oncol* **2012**, *138*, 573-584.
24. Zheng, Y.S.; Zhang, H.; Zhang, X.J.; Feng, D.D.; Luo, X.Q.; Zeng, C.W.; Lin, K.Y.; Zhou, H.; Qu, L.H.; Zhang, P., *et al.* Mir-100 regulates cell differentiation and survival by targeting rbsp3, a phosphatase-like tumor suppressor in acute myeloid leukemia. *Oncogene* **2012**, *31*, 80-92.
25. Zheng, B.; Liang, L.; Wang, C.; Huang, S.; Cao, X.; Zha, R.; Liu, L.; Jia, D.; Tian, Q.; Wu, J., *et al.* Microrna-148a suppresses tumor cell invasion and metastasis by downregulating rock1 in gastric cancer. *Clin Cancer Res* **2011**, *17*, 7574-7583.
26. Wang, B.; Li, W.; Liu, H.; Yang, L.; Liao, Q.; Cui, S.; Wang, H.; Zhao, L. Mir-29b suppresses tumor growth and metastasis in colorectal cancer via downregulating tiam1 expression and inhibiting epithelial-mesenchymal transition. *Cell Death Dis* **2014**, *5*, e1335.
27. Michael, M.Z.; SM, O.C.; van Holst Pellekaan, N.G.; Young, G.P.; James, R.J. Reduced accumulation of specific micrornas in colorectal neoplasia. *Mol Cancer Res* **2003**, *1*, 882-891.
28. Wu, X.L.; Cheng, B.; Li, P.Y.; Huang, H.J.; Zhao, Q.; Dan, Z.L.; Tian, D.A.; Zhang, P. Microrna-143 suppresses gastric cancer cell growth and induces apoptosis by targeting cox-2. *World J Gastroenterol* **2013**, *19*, 7758-7765.
29. Sun, Q.; Zhang, J.; Cao, W.; Wang, X.; Xu, Q.; Yan, M.; Wu, X.; Chen, W. Dysregulated mir-363 affects head and neck cancer invasion and metastasis by targeting podoplanin. *Int J Biochem Cell Biol* **2013**, *45*, 513-520.
30. Pekarsky, Y.; Santanam, U.; Cimmino, A.; Palamarchuk, A.; Efanov, A.; Maximov, V.; Volinia, S.; Alder, H.; Liu, C.G.; Rassenti, L., *et al.* Tcl1 expression in chronic lymphocytic leukemia is regulated by mir-29 and mir-181. *Cancer Res* **2006**, *66*, 11590-11593.
31. Xu, N.; Zhang, L.; Meisgen, F.; Harada, M.; Heilborn, J.; Homey, B.; Grander, D.; Stahle, M.; Sonkoly, E.; Pivarcsi, A. Microrna-125b down-regulates matrix metallopeptidase 13 and inhibits cutaneous squamous cell carcinoma cell proliferation, migration, and invasion. *J Biol Chem* **2012**, *287*, 29899-29908.
32. Chen, Z.L.; Zhao, X.H.; Wang, J.W.; Li, B.Z.; Wang, Z.; Sun, J.; Tan, F.W.; Ding, D.P.; Xu, X.H.; Zhou, F., *et al.* Microrna-92a promotes lymph node metastasis of human esophageal squamous cell carcinoma via e-cadherin. *J Biol Chem* **2011**, *286*, 10725-10734.

33. Lefort, K.; Brooks, Y.; Ostano, P.; Cario-Andre, M.; Calpini, V.; Guinea-Viniegra, J.; Albinger-Hegyi, A.; Hoetzenecker, W.; Kolfschoten, I.; Wagner, E.F., *et al.* A mir-34a-sirt6 axis in the squamous cell differentiation network. *EMBO J* **2013**, *32*, 2248-2263.
34. Zhang, H.; Zuo, Z.; Lu, X.; Wang, L.; Wang, H.; Zhu, Z. Mir-25 regulates apoptosis by targeting bim in human ovarian cancer. *Oncol Rep* **2012**, *27*, 594-598.
35. Zhao, S.; Yao, D.; Chen, J.; Ding, N.; Ren, F. Mir-20a promotes cervical cancer proliferation and metastasis in vitro and in vivo. *PLoS One* **2015**, *10*, e0120905.
36. Lin, C.W.; Chang, Y.L.; Chang, Y.C.; Lin, J.C.; Chen, C.C.; Pan, S.H.; Wu, C.T.; Chen, H.Y.; Yang, S.C.; Hong, T.M., *et al.* Microrna-135b promotes lung cancer metastasis by regulating multiple targets in the hippo pathway and lzts1. *Nat Commun* **2013**, *4*, 1877.
37. Moriarty, C.H.; Pursell, B.; Mercurio, A.M. Mir-10b targets tiam1: Implications for rac activation and carcinoma migration. *J Biol Chem* **2010**, *285*, 20541-20546.
38. Zhang, W.; Zou, C.; Pan, L.; Xu, Y.; Qi, W.; Ma, G.; Hou, Y.; Jiang, P. Microrna-140-5p inhibits the progression of colorectal cancer by targeting vegfa. *Cell Physiol Biochem* **2015**, *37*, 1123-1133.
39. Catuogno, S.; Cerchia, L.; Romano, G.; Pognonec, P.; Condorelli, G.; de Franciscis, V. Mir-34c may protect lung cancer cells from paclitaxel-induced apoptosis. *Oncogene* **2013**, *32*, 341-351.
40. Venkataraman, S.; Birks, D.K.; Balakrishnan, I.; Alimova, I.; Harris, P.S.; Patel, P.R.; Handler, M.H.; Dubuc, A.; Taylor, M.D.; Foreman, N.K., *et al.* Microrna 218 acts as a tumor suppressor by targeting multiple cancer phenotype-associated genes in medulloblastoma. *J Biol Chem* **2013**, *288*, 1918-1928.
41. Li, F.; Li, X.J.; Qiao, L.; Shi, F.; Liu, W.; Li, Y.; Dang, Y.P.; Gu, W.J.; Wang, X.G.; Liu, W. Mir-98 suppresses melanoma metastasis through a negative feedback loop with its target gene il-6. *Exp Mol Med* **2014**, *46*, e116.
42. Fei, J.; Li, Y.; Zhu, X.; Luo, X. Mir-181a post-transcriptionally downregulates oncogenic rala and contributes to growth inhibition and apoptosis in chronic myelogenous leukemia (cml). *PLoS One* **2012**, *7*, e32834.
43. Zhang, X.; Nie, Y.; Li, X.; Wu, G.; Huang, Q.; Cao, J.; Du, Y.; Li, J.; Deng, R.; Huang, D., *et al.* Microrna-181a functions as an oncomir in gastric cancer by targeting the tumour suppressor gene atm. *Pathol Oncol Res* **2014**, *20*, 381-389.
44. Ciafre, S.A.; Galardi, S.; Mangiola, A.; Ferracin, M.; Liu, C.G.; Sabatino, G.; Negrini, M.; Maira, G.; Croce, C.M.; Farace, M.G. Extensive modulation of a set of micrornas in primary glioblastoma. *Biochem Biophys Res Commun* **2005**, *334*, 1351-1358.
45. Ji, D.; Chen, Z.; Li, M.; Zhan, T.; Yao, Y.; Zhang, Z.; Xi, J.; Yan, L.; Gu, J. Microrna-181a promotes tumor growth and liver metastasis in colorectal cancer by targeting the tumor suppressor wif-1. *Mol Cancer* **2014**, *13*, 86.
46. Nagpal, N.; Ahmad, H.M.; Molparia, B.; Kulshreshtha, R. Microrna-191, an estrogen-responsive microrna, functions as an oncogenic regulator in human breast cancer. *Carcinogenesis* **2013**, *34*, 1889-1899.
47. Zhu, D.; Chen, H.; Yang, X.; Chen, W.; Wang, L.; Xu, J.; Yu, L. Mir-32 functions as a tumor suppressor and directly targets sox9 in human non-small cell lung cancer. *Onco Targets Ther* **2015**, *8*, 1773-1783.
48. Humphreys, K.J.; McKinnon, R.A.; Michael, M.Z. Mir-18a inhibits cdc42 and plays a tumour suppressor role in colorectal cancer cells. *PLoS One* **2014**, *9*, e112288.
49. Tsang, W.P.; Kwok, T.T. The mir-18a* microrna functions as a potential tumor suppressor by targeting on k-ras. *Carcinogenesis* **2009**, *30*, 953-959.
50. Cimmino, A.; Calin, G.A.; Fabbri, M.; Iorio, M.V.; Ferracin, M.; Shimizu, M.; Wojcik, S.E.; Aqeilan, R.I.; Zupo, S.; Dono, M., *et al.* Mir-15 and mir-16 induce apoptosis by targeting bcl2. *Proc Natl Acad Sci U S A* **2005**, *102*, 13944-13949.

51. Bischoff, A.; Huck, B.; Keller, B.; Strotbek, M.; Schmid, S.; Boerries, M.; Busch, H.; Muller, D.; Olayioye, M.A. Mir149 functions as a tumor suppressor by controlling breast epithelial cell migration and invasion. *Cancer Res* **2014**, *74*, 5256-5265.
52. Xia, X.; Zhang, K.; Cen, G.; Jiang, T.; Cao, J.; Huang, K.; Huang, C.; Zhao, Q.; Qiu, Z. Microrna-301a-3p promotes pancreatic cancer progression via negative regulation of smad4. *Oncotarget* **2015**, *6*, 21046-21063.
53. Fang, Y.; Xue, J.L.; Shen, Q.; Chen, J.; Tian, L. Microrna-7 inhibits tumor growth and metastasis by targeting the phosphoinositide 3-kinase/akt pathway in hepatocellular carcinoma. *Hepatology* **2012**, *55*, 1852-1862.
54. Yu, T.; Li, J.; Yan, M.; Liu, L.; Lin, H.; Zhao, F.; Sun, L.; Zhang, Y.; Cui, Y.; Zhang, F., *et al.* Microrna-193a-3p and -5p suppress the metastasis of human non-small-cell lung cancer by downregulating the erbb4/pik3r3/mtor/s6k2 signaling pathway. *Oncogene* **2015**, *34*, 413-423.
55. Jin, M.; Zhang, T.; Liu, C.; Badeaux, M.A.; Liu, B.; Liu, R.; Jeter, C.; Chen, X.; Vlassov, A.V.; Tang, D.G. Mirna-128 suppresses prostate cancer by inhibiting bmi-1 to inhibit tumor-initiating cells. *Cancer Res* **2014**, *74*, 4183-4195.
56. Majid, S.; Dar, A.A.; Saini, S.; Deng, G.; Chang, I.; Greene, K.; Tanaka, Y.; Dahiya, R.; Yamamura, S. Microrna-23b functions as a tumor suppressor by regulating zeb1 in bladder cancer. *PLoS One* **2013**, *8*, e67686.
57. Wang, H.; Zhang, W.; Zuo, Y.; Ding, M.; Ke, C.; Yan, R.; Zhan, H.; Liu, J.; Wang, J. Mir-9 promotes cell proliferation and inhibits apoptosis by targeting lass2 in bladder cancer. *Tumour Biol* **2015**, *36*, 9631-9640.
58. Sonkoly, E.; Loven, J.; Xu, N.; Meisgen, F.; Wei, T.; Brodin, P.; Jaks, V.; Kasper, M.; Shimokawa, T.; Harada, M., *et al*. Microrna-203 functions as a tumor suppressor in basal cell carcinoma. *Oncogenesis* **2012**, *1*, e3.
